# Purification and characterisation of glutathione reductase from scorpionfish *(scorpaena porcus)* and investigation of heavy metal ions inhibition

**DOI:** 10.1080/14756366.2023.2167078

**Published:** 2023-03-20

**Authors:** Kübra Işık, Ercan Soydan

**Affiliations:** Department of Agricultural Biotechnology, Faculty of Agriculture, Ondokuz Mayıs University, Samsun, Turkey

**Keywords:** Glutathione reductase, scorpion fish, enzyme purification, metal ion, inhibition

## Abstract

In the current study, glutathione reductase was purified from Scorpion fish (*Scorpaena porcus*) liver tissue and the effects of heavy metal ions on the enzyme activity were determined. The purification process consisted of three stages; preparation of the homogenate, ammonium sulphate precipitation and affinity chromatography purification. At the end of these steps, the enzyme was purified 25.9-fold with a specific activity of 10.479 EU/mg and a yield of 28.3%. The optimum pH was found to be 6.5, optimum substrate concentration was 2 mM NADPH and optimum buffer was 300 mM KH_2_PO_4._ After purification, inhibition effects of Mn^+2^, Cd^+2^, Ni^+2^, and Cr^3+^, as heavy metal ions were investigated. IC_50_ values of the heavy metals were calculated as 2.4 µM, 30 µM, 135 µM and 206 µM, respectively.

## Introduction

Scorpionfish (*Scorpaena porcus*), a member of the Scorpaenidae family, is most prevalent in Mediterranean and Black Sea regions, prefering shallow, algae-covered regions at depths of up to 1000 m.^1^ Pollution in the sea mainly accumulates in marine organisms and sediments. Therefore, it is transmitted to humans through the food chain^2^. In the aquatic ecology, fish have the greatest trophic level.^3^ Pollutants such as heavy metals, environmental and industrial wastes mix with the waters and may accumulate in fish. Heavy metals, which are one of the most significant polluting factors, have negative impacts on human health and may induce production of reactive oxygen species (ROS) resulting in oxidative stress. Reactive oxygen species cause damage to cell components such as lipids, proteins, and DNA^4^. As a result, antioxidants are the most significant agents in removing oxidative damage generated by ROS in the live body^5^.

Antioxidants are known as important member of defense system preventing formation of reactive oxygen species and repairing the damage they cause^6^. The continuity of life of living cells depends on the balance of complex biochemical reactions. Endogenous/exogenous compounds arising from the factors that will disrupt this balance cause cell destruction^7^.

Glutathione, a natural reducing molecule, may be easily employed by cells to protect themselves against oxidative stress. This protective effect against ROS is provided by interaction with enzymes such as glutathione peroxidase and glutathione reductase^8^. In addition to being an antioxidant, GSH has a role in the detoxification system of the cell, gene expression and regulation^9^.

Glutathione reductase (EC 1.8.1.7; GR), a major enzyme in glutathione metabolism, is required for the maintenance of the reduced form of cellular glutathione, which is strongly nucleophilic for many reactive electrophiles^10^^,^^11^. The flavin enzyme GR acts as an antioxidant to protect cells from oxidative stress by reducing glutathione disulphide (GSSG) to its reduced form (GSH)^12^. It has an important role in the drug and detoxification mechanisms especially in the liver. This is due to the cytochrome P-450 system found in liver microsomes, which provides detoxifying events^13^. Maintaining the GSH/GSSG ratio in the cell environment is one of the most important known targets of the GR enzyme-catalysed reactions^14^. Glutathione reductase is involved in the reduction-oxidation of intracellular glutathione for GSSG, which is generated through the detoxification of hydroperoxides and reduction of some other chemicals catalysed by glutathione perdoxidase^15^. The NADP^+^ dependent malate dehydrogenase and pentose phosphate pathways provide the NADPH needed in this catalytic process^16^^,^^17^. NADPH, a key product of the pentose phosphate cycle, is employed extensively in reductive biosynthesis. Furthermore, it aids in the protection of the cell against oxidative damage^9^.

A lack of GR and GSH causes oxidative damage to the cell. Many disorders are caused by GR and GSH deficiencies, including Alzheimer’s, Parkinson’s, liver and lung diseases, sickle cell anaemia, HIV, AIDS, cancer, stroke, schizophrenia, and diabetes^9^^,^^18^.

Metals found naturally in the environment and in water originating from natural and anthropogenic causes. Several metals and chemicals have been tested on various enzymes for their inhibitory effects^19^. Due to many industrial problems, heavy metal accumulation in the environment has become an important problem^20^. Heavy metals, which can be harmful even at low quantities, enter the body through the mouth, breathing and skin. They cannot be eliminated from the excretory tracts such as kidney, liver, intestine, lung and skin without special intervention. Consequently, almost all heavy metals accumulate in biological organisms. These metals build up in living things and cause major disorders such thyroid neurological diseases, autism and infertility. They trigger and enhance the generation of free radicals in aquatic species. Therefore, it should be kept under control in order to prevent the damage caused by free radicals resulting from heavy metals or chemicals^21–25^. Heavy metals and metal ions have an effect on the variables that affect enzyme-substrate and cofactor affinity. Metal ions, which induce transient depletion of GSH and inhibition of antioxidant enzymes, are known to have an effect on enzyme function^26^. It is also known that the glutathione reductase enzyme is highly sensitive to metal ions when the GSSG concentration is low^27^.

Therefore, we aimed in this study to purify and characterise GR enzyme from the liver tissue of scorpion fish for the first time and evaluate the inhibitory effects of heavy metals, namely Mn^+2^, Cd^+2^, Ni^+2^, and Cr^3+^. The reason for investigating scorpion fish enzyme is that the fish is commonly found in our region and the purpose of selection of these metals is the fact that they are among the most common metals found in our seas and rivers as industrial and environmental waste. Thus, the risk of these metal ions should be well characterised as our seas and water sources are at great risk of metal pollution.

## Materials and methods

### Chemicals

All chemicals used for the purification process were used from Sigma-Aldrich. All other analytical grade chemicals were obtained from Merck.

### Preparation of the homogenate

Scorpion fish (*Scorpaena porcus*) were collected from a fisherman in Ordu’s Fatsa region, Turkey. 7.5 g of the liver tissues taken were weighed and homogenised in liquid nitrogen in a mortar. 40 ml of 50 mM KH_2_PO_4_ + 1 mM EDTA + 1 mM DTT + 1 mM PMSF buffer was poured to a 50 ml falcon tube. The samples were then centrifuged for 60 min at +4 °C at 27000 g. The supernatant and precipitate were separated from the filter paper after centrifugation, and the enzyme activity was measured.

### Ammonium sulphate precipitation and dialysis

Ammonium sulphate ((NH_4_)_2_SO_4_) precipitation was performed for the separation of the relevant protein from other proteins or for the concentration of the proteins. Scorpion fish liver tissue extract was subjected to a precipitation range of 0–100%. As a result of the process, the enzyme’s active range was determined by achieving saturation in the 60–80% range. The precipitate was dissolved in a buffer of KH_2_PO_4_ (300 mM; pH:6.5). After the specified interval, dialysis was performed three times to desalinate the protein solution. It was then dialysed for 2 h in a solution of 30 mM KH_2_PO_4_ (pH: 6.5).

#### 2′,5′-Adp Sepharose-4B affinity chromatography

2 grams of dried 2,5-ADP Sepharose-4B was used for a 10 ml bed volume column (1 × 10 cm). To eliminate foreign bodies and air, the gel was rinsed with 300 ml of distilled water. It was packaged in colas after being suspended in an equilibration solution of 50 mM K_2_PO_4_ + 1 mM EDTA + 1 mM DTT pH: 7.3. It was understood that the column was equilibrated by equalising the absorbance and pH of the eluate and buffer at 280 nm. The column was loaded with a sample that had been precipitated in the indicated ammonium sulphate saturation range and dialysed. The column was continued to wash with 0.1 M + K-acetate + 0.1 M K-phosphate pH: 7.85 and 0.1 M K-phosphate + 0.1 M KCl pH: 7.85 buffer. 50 mM K-phosphate + 1 mM EDTA + 1 mM GSH + 0.5 M mM NADPH pH:7.3 was used to elute the enzyme. Elutions were collected and eluted on the column with 01. M Na-acetate + 0.5 M NaCl pH: 4.5, 0.1 M Tris + 0.5 NaCl pH: 8.5 regeneration buffers. All these procedures were performed at 4 °C.

### Protein determination

Protein content was determined spectrophotometrically at 595 nm using Bradford’s technique using bovine serum albumin as a reference for all samples^28^. The µg protein values corresponding to the absorbance values from the results obtained were turned into a standard graph.

### SDS polyacrylamide gel electropheresis (SDS-PAGE)

The Laemmle technique was used to assess the purity of the enzymes^29^. For the separating gel and stacking gel, concentrations of 30% acrylamide-0.8% bisacrylamide were used in the gel technique. 10% SDS (final concentration: 0.01%) was added to the gel solution. The gel was stabilised in a solution containing 40% methanol + 10% acetic acid + 80% distilled water for 1 h. For around 45 min, staining was done in a 40% methanol + 10% acetic acid + 50% distilled water + 0.25% Coommassie Brilliant Blue R-250 solution. The gel was rinsed in 10% methanol + 10% acetic acid + 80% distilled water at the end of the colouring procedure. The gel was left in the wash solution for one day to clean the protein bands.

### *In vitro* effects of metal ions

To assess the impact of metal ions on Scorpion Liver GR, an inhibition research was carried out. The effects of heavy metals on GR enzyme were studied at Ni^2+^ (0.001 M), Mn^2+^ (0.001 M), Cr^3+^ (0.001 M) and Cd^2+^ (0.001 M) concentrations using the salts Ni(NO_3_)_2_, Mn(NO_3_)_2_ Cr(NO_3_)_3_ Cd(NO_3_)_2_. Activity values were used by plotting the % activity- [metal ion] graphs (Figures 2, 3 and 4, Supplementary Material). Metal concentrations producing 50% inhibition of enzyme activity (IC50) were determined. The The IC_50_ values of heavy metals were calculated as 2.4 µM, 30 µM, 135 µM and 206 µM, for Mn^+2^, Cd^+2^, Ni^+3^, and Cr^+^, respectively (Table 1).

**Figure 1.**
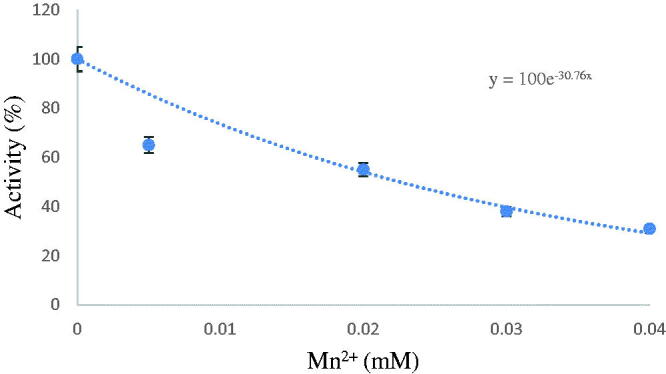
Activity %-inhibitor regression analysis graph for Scorpionfish GR in the presence of different Mn^2+^ concentrations.

**Table 1.** Scorpionfish GR enzyme inhibition data by heavy metal ions.

Metal ion	IC_50_ (µM)
Ni^2+^	135 ± 1.9
Mn^2+^	2.4 ± 0.04
Cr^3+^	206 ± 3.01
Cd^2+^	30 ± 0.4

## Results and discussion

Glutathione (GSH), a tripeptide that is synthesised in the liver, is present in the cytosol, nucleus and mitochondria of cells and plays vital roles in the cell^30^^,^^31^. Glutathione reductase catalyses electron transfer between low or high molecular weight disulphide substrates and reduced pyridine nucleotides, which is one of the essential enzymes of the intracellular antioxidant system that protects cells from the detrimental effects of free radicals^15^. The reduction of glutathione disulphide (GSSG) by means of NADPH is catalysed by GR^32^. The most important goal of the GR enzyme-catalysed reaction is to keep the GSH/GSSG ratio in the cell environment stable^14^. It not only maintains the GSH/GSSG ratio, but also supports the continuance of the cell’s important tasks, such as detoxification of ROS^32^. There is a balance between free radicals and the antioxidant defense system. Overproduction of free radicals causes harm to antioxidant defense mechanisms. Therefore, the cell is exposed to oxidative stress as a result of the balance between free radicals and the antioxidant system^9^.

GSH, which preserves proteins in their reduced state, contributes to the spherical structure of erythrocytes. Erythrocytes that are susceptible to oxidative damage live shorter as a result of GSH depletion, resulting in haemolytic anemia^33^. Asnis was the first to purify the glutathione reductase enzyme from Escherichia coli^34^. It has been purified and characterised from mammalian tissues such as human erythrocytes, bovine erythrocytes, bovine liver, turtle, chicken liver, rat liver, bovine brain, plants such as pea leaves, and many bacterial species in numerous other studies^10^^,^^35–41^.

Heavy metals are formed today as a result of fast population expansion, urban garbage, industrial waste, and unintentionally used fertilisers and pesticides in agriculture. These heavy metals, which mix with soil, sea and rivers, cause some problems in organisms due to the inhibition of antioxidant enzymes in the cell and oxidative damage by the exposure of living things.

In this study, glutathione reductase enzyme was isolated from the liver tissue of a scorpion fish (Scorpaena porcus) and some of its kinetic features were investigated. Purification methods were performed by homogenate preparation, ammonium sulphate precipitation, dialysis, 2′,5′-ADP Sepharose-4B affinity chromatography and SDS polyacrylamide gel electrophoresis (SDS-PAGE), respectively.

The purification process began with the homogenate preparation step. Ammonium sulphate precipitations in the range of 0–100% were performed on the prepared liver tissue. The GR enzyme precipitated in the intervals of 60–80% during the precipitation procedure. After precipitation, dialysis was performed to remove ions in the medium before affinity chromatography. Following dialysis, purification was performed on a 2′,5′-ADP Sepharose 4B affinity column, and the molecular weight of GR was determined using the SDS polyacrylamide gel electrophoresis. The liver tissue was purified 25.96 times with a yield of 28.277% and its molecular weight was determined to be 25 kDa. Quantitative protein determination was determined by the Bradford method. Ni^2+^, Mn^2+,^ Cr^3+^, and Cd^2+^ heavy metals were applied on the purified enzyme. IC_50_ values of heavy metals were calculated as 2.4 µM, 30 µM, 135 µM and 206 µM, for Mn^2+^, Cd^2+^, Ni^2+^, and Cr^3+^, respectively.

The characterisation process was also carried out in the study. For this purpose, optimum pH, optimum substrate and optimum buffer values of GR enzyme were determined from scorpion fish liver. The optimum pH of the GR enzyme was found to be 6.5, the optimum substrate was 2 mM NADPH and the optimum buffer was 300 mM KH_2_PO_4_.

Many studies have been carried out on glutathione reductase enzyme including purification from many tissues, characterisation and determination of biochemical properties.

Erat and Çiftçi (2006) purified glutathione reductase enzyme 5.823 times from human erythrocytes with 24% efficiency in their study^42^. Senturk et al. (2008) isolated human erythrocyte glutathione reductase enzyme 2555.56 fold with 29.74% yield^43^. Akkemik et al. (2011) analysed the effects of some drugs on glutathione reductase from human erythrocytes, they purified the GR enzyme 3333 fold with a yield of 44.44%^44^. Ulusu and Tandoğan (2007) purified GR enzyme 5456 fold with 38.4% yield in their study from beef liver^40^. Senturk et al. (2009) investigated the effects of some analgesic and anestethic drugs on the glutathione reductase enzyme purified from human erythrocytes 2139-fold with a yield of 29^45^. In another study, Taşer and Çiftçi (2012) purified the enzyme from turkey liver 2476 fold with a yield of 10.75%^46^. Ekinci and Şentürk (2013) isolated the enzyme from rainbow trout liver and investigated the effects of Co^+2^, Zn^+2^, Ca^+2^, Fe^+2^, Mn^+2^, Cr^+3^, Sn^+2^ and Mg^2+^. The heavy metals had IC_50_ values of 42.2, 63.1, 357, 486, 508, 592, and 657, respectively^47^. Tekman et al. (2008) also evaluated the influences of Cd^+2^, Cu^+2^, Pb^+2^, Hg^+2^, Fe^+3^ and Al^+3^ on rainbow trout GR enzyme. The IC_50_ values were found to be 65.5, 82, 122, 509, 797 and 804 µM, respectively^48^. Karagozoglu and Ciftci investigated heavy metal inhibition of GR enzyme purified from chicken kidney with 57% efficiency. They determined the inhibition effects of heavy metals Ni^+2^, Zn^+2^, Pb^+2^, Hg^+2^, Ag^+^ and Al^+3^ on the GR enzyme. The IC_50_ values were found to be 337, 191, 168, 187 and 289, respectively^49^.

The influence of different metal ions on GR enzyme isolated from scorpion liver tissue has been analysed in our work. Scorpion fish liver GR enzyme with a specific activity of 10.479 EU/mg was purified 25.96-fold with a yield of 28.277%. When the received data was compared to the literature studies, our results are in accordance with previous works. SDS-PAGE revealed the molecular weight of the enzyme as 25 kDa which is also similar to the literature data.

The relevance of the study is demonstrated by the damage caused by heavy metals in nature. Because heavy metals, which arise as a result of environmental problems, mix with soil, water and seas and damage the living ecosystem. Especially heavy metals accumulating in seas and rivers cause exposure to aquatic organisms. As a result of exposure of aquatic organisms, these heavy metals are transmitted to humans through food intake.

In conclusion, in this study, it was determined that liver tissue GR was inhibited by metal ions in micromolar levels and the strongest inhibitor was Mn^2+.^ The inhibition of GR with various heavy metals has a negative effect on the organism. Therefore, specific care should be taken in the use of these metals, which can disrupt the GSH/GSSG balance by inhibiting the GR enzyme. This is the first research dealing with the purification and characterisation of GR enzyme from Scorpion fish liver tissue. We hope findings of our study will be helpful to further researchers interested in toxicology and enzymology.

## Supplementary Material

Supplemental Material
