# Supplementary material for: Purification and characterisation of glutathione reductase from scorpionfish (scorpaena porcus) and investigation of heavy metal ions inhibition
Source: J Enzyme Inhib Med Chem. 2023 Mar 20;38(1):2167078. doi: 10.1080/14756366.2023.2167078 (PMC10035961; doi:10.1080/14756366.2023.2167078)
Supplement: Supplemental Material [file IENZ_A_2167078_SM7068.pdf]

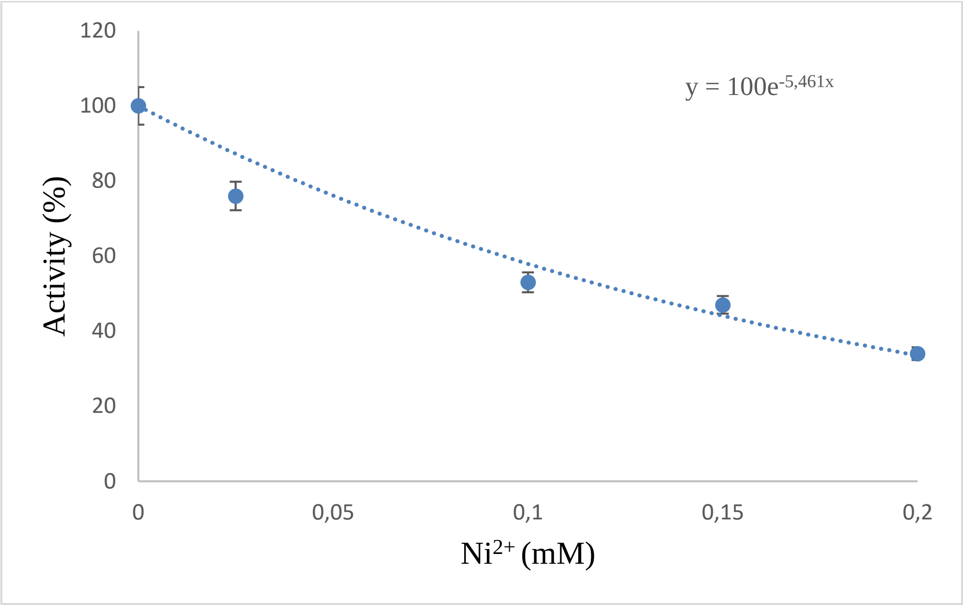

Figure 2: Activity %-inhibitor regression analysis graph for Scorpionfish GR in the presence of different Ni^{2+} concentrations.

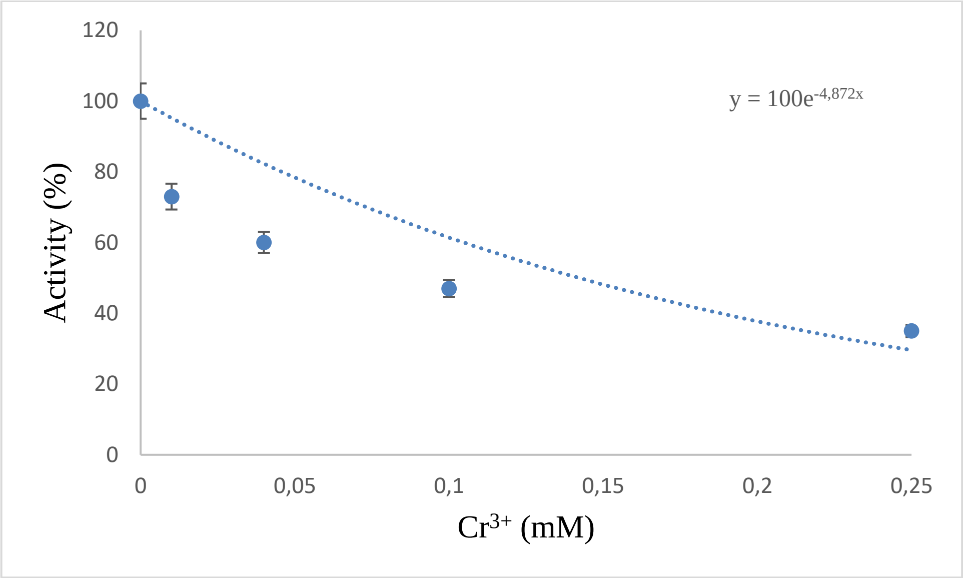

Figure 3: Activity %-inhibitor regression analysis graph for Scorpionfish GR in the presence of different Cr^{3+} concentrations.

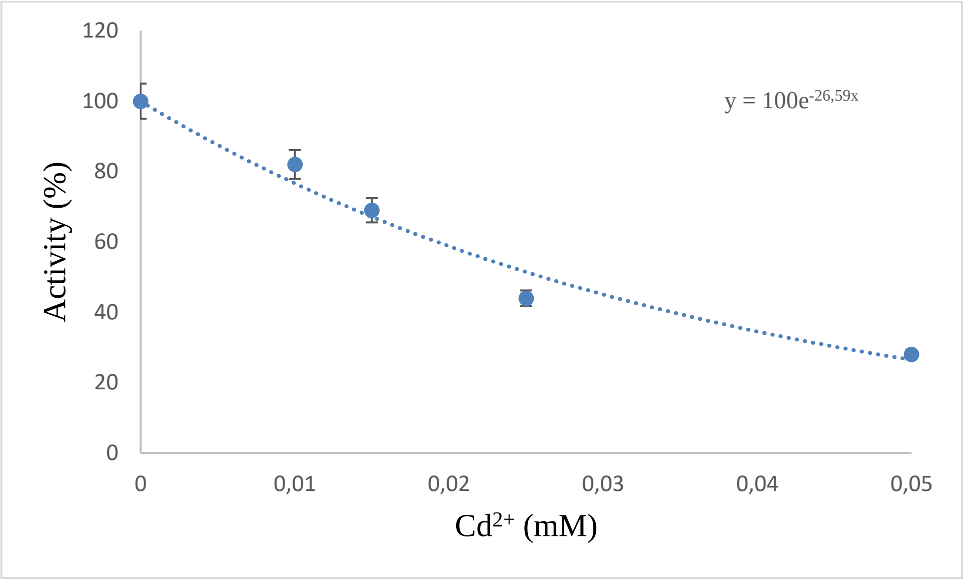

Figure 4: Activity %-inhibitor regression analysis graph for Scorpionfish GR in the presence of different Cd^{2+} concentrations.
